# Diverse and multifunctional roles for perlecan (*HSPG2*) in repair of the intervertebral disc

**DOI:** 10.1002/jsp2.1362

**Published:** 2024-07-29

**Authors:** James Melrose, Farshid Guilak

**Affiliations:** ^1^ Raymond Purves Bone and Joint Research Laboratory, Kolling Institute Northern Sydney Local Health District St. Leonards New South Wales Australia; ^2^ Graduate School of Biomedical Engineering University of New South Wales Sydney New South Wales Australia; ^3^ Sydney Medical School, Northern The University of Sydney St. Leonards New South Wales Australia; ^4^ Faculty of Medicine and Health The University of Sydney, Royal North Shore Hospital St. Leonards New South Wales Australia; ^5^ Department of Orthopaedic Surgery Washington University St. Louis Missouri USA; ^6^ Department of Orthopaedics Shriners Hospitals for Children St. Louis Missouri USA

**Keywords:** chondroid metaplasia, homeostasis, intervertebral disc, intervertebral disc degeneration, mechanotransduction, osmoregulation, perlecan

## Abstract

Perlecan is a widely distributed, modular, and multifunctional heparan sulfate proteoglycan, which facilitates cellular communication with the extracellular environment to promote tissue development, tissue homeostasis, and optimization of biomechanical tissue functions. Perlecan‐mediated osmotic mechanotransduction serves to regulate the metabolic activity of cells in tissues subjected to tension, compression, or shear. Perlecan interacts with a vast array of extracellular matrix (ECM) proteins through which it stabilizes tissues and regulates the proliferation or differentiation of resident cell populations. Here we examine the roles of the HS‐proteoglycan perlecan in the normal and destabilized intervertebral disc. The intervertebral disc cell has evolved to survive in a hostile weight bearing, acidic, low oxygen tension, and low nutrition environment, and perlecan provides cytoprotection, shields disc cells from excessive compressive forces, and sequesters a range of growth factors in the disc cell environment where they aid in cellular survival, proliferation, and differentiation. The cells in mechanically destabilized connective tissues attempt to re‐establish optimal tissue composition and tissue functional properties by changing the properties of their ECM, in the process of chondroid metaplasia. We explore the possibility that perlecan assists in these cell‐mediated tissue remodeling responses by regulating disc cell anabolism. Perlecan's mechano‐osmotic transductive property may be of potential therapeutic application.

## Graphical abstract

Perlecan is a modular multifunctional cell instructive proteoglycan that conveys mechanosensory and osmo‐regulatory properties that are important for the homeostasis of tissue composition and function in the intervertebral disc. Perlecan shows potential in repair biology in weightbearing, tensional tissues, and those resisting shear forces.

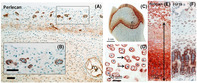

**Abbreviations**
AF: annulus fibrosus
AFM: atomic force microscopy
ANG: angiogenin
BBB: blood brain barrier
CEPs: cartilaginous endplates
CS: chondroitin sulphate
ECM: extracellular matrix
EGF: epidermal growth factor
FAK: focal adhesion kinase
FGF: fibroblast growth factor
GAG: glycosaminoglycan
HA: hyaluronan
HRP: horseradish peroxidase
HS: heparan sulphate
*HSPG2*
: HS proteoglycan 2
HSPGs: HS proteoglycans
IVD: intervertebral disc
IVDD: IVD degeneration
LG: laminin G domain
LBP: low back pain
LumC13: C terminal lumikine domain of lumican
LRR: leucine rich repeat
MMPs: matrix metalloproteinases
NP: nucleus pulposus
NF‐κB: nuclear factor kappa B
OA: osteoarthritis
PDGF: platelet derived growth factor
PCM: pericellular matrix
PGs: proteoglycans
*TRPV4*
: transient receptor potential cation channel subfamily V member 4 ion channel
ROCK: rho kinase
VB: vertebral body
VEGF: vascular endothelial cell growth factor
VLDLR: very low‐density lipoprotein receptor

## 1 INTRODUCTION

### 1.1 Aims of the study

The intervertebral disc (IVD) appears unable to self‐repair in response to experimental IVD degeneration (IVDD). In the course of multiple studies on an ovine large annular lesion model of IVDD, we observed that a proportion of the experimental animals developed chondroid metaplastic deposits, which apparently stabilized this defect and prevented lesion propagation and the development of IVDD. This chondroid metaplasia appeared to develop from chondroid progenitor cells present in the IVD and thus may represent an intrinsic self‐repair response. We hypothesize that, based on its multifunctional properties in cartilaginous tissues, the heparan‐sulfate‐proteoglycan perlecan (*HSPG2*) may play a role in such events. Here, we discuss this hypothesis in light of the large body of literature on perlecan and its function in the IVD.

### 1.2 Perlecan has a widespread distribution in cartilaginous tissues

Perlecan is a ubiquitous component of basement membranes in vascularized tissues. It also has a widespread distribution in the avascular tensional and weight bearing cartilages such as the meniscus, tendon, ligament, and IVD, which are devoid of basement membrane; the chondrocyte pericellular matrix (PCM) may represent an intrinsic basement membrane surrounding cartilaginous cells.^1^ Perlecan is localized in the periphery of stem cell niches in fetal cartilage rudiments^2^ and regulates the attainment of stem cell pluripotency and the development of migratory chondroprogenitor stem cell lineages with roles in tissue development, expansion of cartilage rudiments and development of primary and secondary ossification center precursors to the cartilage growth plate. Atomic force microscopy demonstrates that perlecan imparts compliancy to the PCM and is cytoprotective.^3^ Cell‐extracellular matrix (ECM) interconnections in cartilages provided by perlecan have biosensory osmoregulatory properties, allowing cells to perceive and respond to perturbations in their biomechanical microenvironments and to orchestrate tissue homeostasis. Perlecan also monitors the flow of cannalicular fluid in the osteocyte PCM and acts as a fluid shear biosensor that regulates bone development.^4^, ^5^, ^6^

**FIGURE 1:**
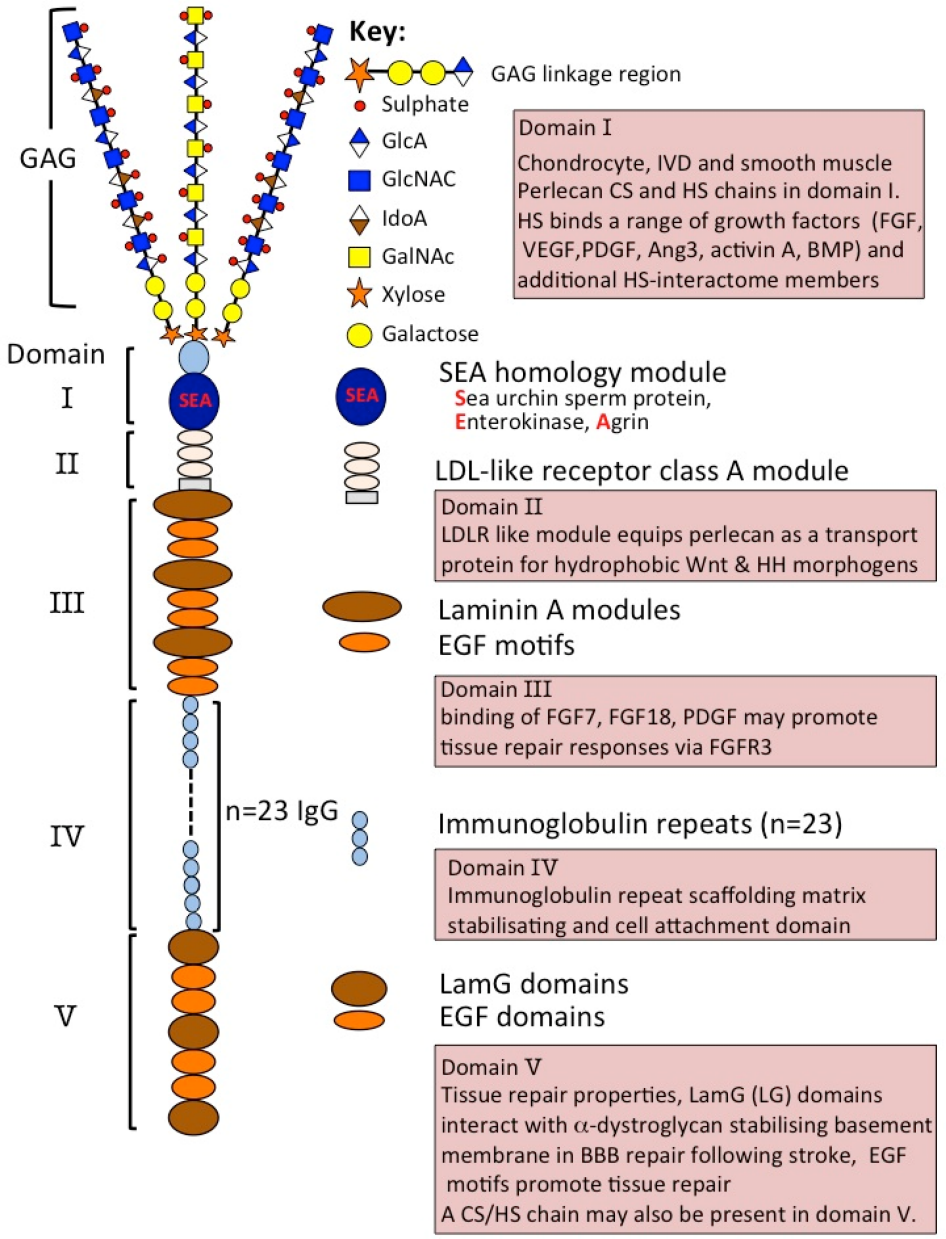
Schematic depiction of the modular organization of perlecan showing its five domains and their bioactive modules and some of the major functional properties of specific domains (boxed comments). The glycosaminoglycan structure of heparan sulfate (HS) and chondroitin sulfate (CS) chains attached to the N‐terminal domain‐I are shown using symbol nomenclature for glycans (SNFGs) icons for glycans. Figure modified from reference 21 with permission.

Perlecan is a large modular multifunctional proteoglycan (PG) with a 467 kDa core protein. Figure 1 depicts the structural organization of perlecan and the functional attributes of each of its five distinct domains. Domain I is unique to perlecan. In perlecan produced by chondrocytes, IVD cells, fibroblasts and smooth muscle cells, Domain I is attached to chondroitin sulfate (CS) and heparan sulfate (HS), whereas endothelial cells produce a monosubstituted perlecan containing HS chains only. These HS side chains bind several members of the fibroblast growth factor (FGF) family, platelet derived growth factor (PDGF), vascular endothelial cell growth factor (VEGF), bone morphogenetic protein (BMP)‐2, 4, angiogenin (Ang)‐3 and activin A to promote cellular proliferation, differentiation and tissue development,^7^ 4,6‐disulphated CS has also been observed to regulate collagen fibrillogenesis introducing a further level of complexity in the regulatory properties of perlecan in cartilaginous tissues.^8^ Perlecan Domain I has been used to deliver these growth factors in tissue repair contexts.^9^, ^10^ Perlecan also has proposed roles in cartilage repair following chondral injury.^11^

Perlecan Domain II bears homology with low‐density lipoprotein (LDL) receptor and has roles in the clearance of LDL and very low‐density lipoprotein (VLDL) from the bloodstream. Domain II binds the poorly soluble Wnt and Hedgehog (Hh) morphogens, allowing perlecan to transport them and aid in the establishment of morphogen gradients important in tissue development.^12^ Domain III of perlecan binds FGF‐7 and 18 through protein–protein and other non‐HS‐mediated interactions and mediates cell proliferation.^13^ Domain IV, a 23 immunoglobulin (Ig) repeat domain, bears homology with the cell membrane Ig receptor family and neural cell adhesion molecule (NCAM) and has roles as a scaffolding material providing tissue stabilization through cell adhesion and self‐aggregative properties. Perlecan domain IV also supports cell spreading through focal adhesion kinase (FAK) activation.^14^

Perlecan domain V contains three laminin‐type G (LG) domains and four epidermal growth factor (EGF)‐like repeats.^15^ The LG domains are homologous with the α chain globular domains of laminin and facilitate cell‐ECM interactions, as well as playing other roles in angiogenesis, vascular cell interactions, wound healing and autophagy.^7^, ^15^, ^16^, ^17^ LG1LG2 and LG3 fragments interact with α2β1 integrin disturbing the assembly of angiogenic capillary tubes. Domain V plus a portion of Domain IV support endothelial cell interactions as effectively as full‐length perlecan when expressed in HEK293 cells.^15^, ^18^

### 1.3 Perlecan is a multifunctional proteoglycan interactive with a diverse range of ligands

The HS side chains of perlecan equip it with an ability to interact with a diverse range of ligands (Table 1) of importance in cartilage development and ECM remodeling, in tissue morphogenesis, and in tissue repair responses.^11^, ^21^, ^22^, ^25^, ^26^, ^27^, ^28^, ^29^, ^30^ This establishes perlecan as a PG of some importance in the development and stabilization of IVD tissues equipping IVD cells with cell‐matrix communicative properties that sense perturbations in their biomechanical environment allowing the IVD cell to orchestrate tissue homeostasis and the maintenance of IVD function.^3^, ^31^

**TABLE 1:** Perlecan‐interactive ligands.

Domain‐I	Domain‐II	Domain‐III	Domain‐IV	Domain‐V
Laminin‐1	VLDL	FGF‐7, 18	Nidogen‐1, 2	Nidogen‐1
Collagen IV, V, VI, XI	LDL	FGFBP	Fibronectin	Fibulin‐2
Fibronectin	Fibrillin‐1	WARP	Collagen IV, VI	β1‐integrin
PRELP, WARP	Wnt, Hh	Collagen VI	PDGF	α‐DG
Fibrillin‐1		Tropoelastin	Fibulin‐2	FGF‐7
Thrombospondin			Tropoelastin	Endostatin
FGF‐1, 2, 7, 9, 10, 18			NG2/CSPG4	ECM‐1
BMP‐2, 4				Collagen VI
PDGF, VEGF, IL2				Progranulin
Hh, Ang‐3				Acetylcholinesterase
Heparanase				α2β1‐integrin
Activin A, Histone H1				Tropoelastin
G6b‐B‐R				NG2/CSPG4

*Note*: Data compiled from references 7, 12, 13, 19, 20, 21, 22, 23, 24. Table adapted from reference 25 with permission.

Abbreviations: Ang‐3, angiogenin like protein‐3; BMP, bone morphogenetic protein; CSPG4, melanoma‐associated chondroitin sulfate proteoglycan, or neuron‐glial antigen 2; ECM‐1, ECM protein‐1; FGF, fibroblast growth factor; G6b‐B‐R, megakaryocyte lineage‐specific immunoreceptor tyrosine‐based inhibition motif–containing receptor; IL, interleukin; PDGF, platelet derived growth factor; PRELP, proline/arginine‐rich end leucine‐rich repeat protein, Prolargin; VEGF, vascular cell endothelial cell growth factor; WARP, von Willebrand factor A domain‐related protein; α‐DG, alpha dystroglycan.

An informatics analysis of the HS binding proteins using the KEGG: Kyoto Encyclopedia of Genes and Genomes shows that HS is implicated in many physiological processes and biological pathways (Table 2) of importance in tissue repair.^32^

**TABLE 2:** The biodiverse processes and biological pathways of HS binding proteins identified in the HS interactome.

(A) GO biological process terms enriched in the heparin/HS interactome.			
Term	Name	Count^a^	%^a^
GO: 0009611	Response to wounding	120	27.8
GO: 0042330	Taxis	55	12.8
GO: 0006935	Chemotaxis	55	12.8
GO: 0006954	Inflammatory response	73	16.9
GO: 0006952	Defense response	91	21.1
GO: 0007626	Locomotory behavior	62	14.4
GO: 0006955	Immune response	91	21.1
GO: 0042060	Wound healing	51	11.8
GO: 0016477	Cell migration	57	13.2
GO: 0007610	Behavior	71	16.5
GO: 0051674	Localization of the cell	58	13.5
GO: 0048870	Cell motility	58	13.5
GO: 0042127	Regulation of cell proliferation	90	20.9
GO: 0006928	Cell motion	70	16.2
GO: 0032101	Regulation of response to external stimulus	43	10.0
GO: 0001568	Blood vessel development	51	11.8
GO: 0001944	Vascular development	51	11.8
GO: 0051605	Protein maturation by peptide bond cleavage	33	7.7
GO: 0007267	Cell–cell signaling	76	17.6
GO: 0016485	Protein processing	36	8.4

(B) KEGG pathways enriched in the heparin/HS interactome.			
Term	Name	Count^a^	%^a^
hsa04610	Complement and coagulation cascades	42	9.7
hsa04060	Cytokine‐cytokine receptor interaction	63	14.6
hsa04512	ECM‐receptor interaction	35	8.1
hsa04510	Focal adhesion	43	10.0
hsa05200	Pathways in cancer	52	12.1
hsa05218	Melanoma	22	5.1
hsa04062	Chemokine signaling pathway	34	7.9
hsa05020	Prion diseases	15	3.5
hsa04810	Regulation of actin cytoskeleton	33	7.7
hsa04350	TGF‐β signaling pathway	18	4.2
hsa04672	Intestinal immune network for IgA production	13	3.0
hsa05322	Systemic lupus erythematosus	18	4.2
hsa04010	MAPK signaling pathway	30	7.0
hsa04640	Hematopoietic cell lineage	14	3.2
hsa04621	NOD‐like receptor signaling pathway	11	2.6
hsa05219	Bladder cancer	9	2.1
hsa05310	Asthma	7	1.6
hsa05222	Small cell lung cancer	12	2.8

*Note*: Data modified from reference 32.

Abbreviations: HS, heparan sulfate; KEGG: Kyoto Encyclopedia of Genes and Genomes.

^a^ Count = number of HS binding proteins; % = percentage of the identified proteins.

**FIGURE 2:**
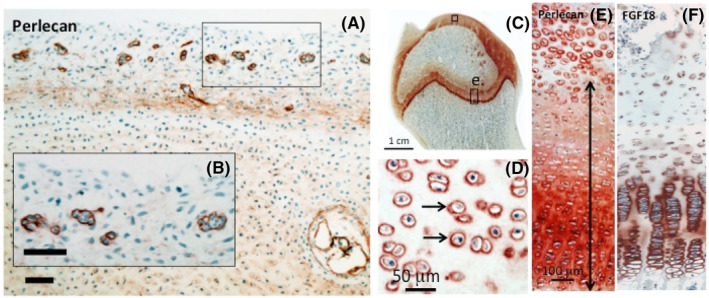
Demonstration of the widespread distribution of perlecan in cartilaginous tissues by immunolocalization using MAb A7L6 rat monoclonal anti‐perlecan domain‐IV antibodies. Immunolocalization in the periphery of chondroprogenitor stem cell niches in a 12‐week‐old human fetal hip rudiment (A). The boxed area in (A) is shown at higher magnification in (B). Macroscopic view of an immature ovine hip joint (C). Boxed areas (D) and (E) depict areas of articular cartilage and growth plate cartilage depicted at higher magnification in (D) and (E). Perlecan is a prominent pericellular matrix proteoglycan of articular chondrocytes and also forms an extracellular gradient in growth plate cartilage (double headed arrow). FGF‐18 interacts with perlecan and is prominently immunolocalized pericellularly around hypertrophic columnar chondrocytes in the growth plate (F). The chromogen used in these bright‐field images was NovaRED. Images (A, B) reproduced from reference 2. Images (C–F) reproduced from reference 21 with permission.

As Figure 2 illustrates, perlecan is a component of progenitor stem cell niches in fetal cartilage rudiments (Figure 2A, B) and is also a prominent pericellular PG of articular (Figure 2D) and growth plate (Figure 2E) chondrocytes during skeletal development. Perlecan forms an extracellular gradient in growth plate cartilage, hypertrophic chondrocyte perlecan binds FGF‐18 (Figure 2F) which promotes cartilage development and endochondral ossification and extension of the axial and appendicular skeleton.^21^, ^22^, ^33^, ^34^, ^35^ Perlecan also has biomechanical roles in the cartilage PCM.^36^

The IVD is a composite fibrocartilaginous connective tissue, which conveys major weight bearing properties and flexibility to the spinal column.^37^ The adult human spine contains 7 cervical, 12 thoracic, 5 lumbar, and 1 sacral IVDs which collectively occupy a third of the total spinal length.^37^ A healthy IVD transmits, redistributes, and dissipates axial spinal biomechanical forces, providing mechanical stability and flexibility during flexion‐extension and torsional rotational movements of the spine. The lumbar and cervical IVDs are the most flexible regions of the spine.

The IVD is composed of an aggrecan PG‐rich central nucleus pulposus (NP), equipping the IVD with weight‐bearing properties when the spine undergoes axial compression.^38^, ^39^ The Gibbs‐Donnan effect due to GAG sulphation and ionizable carboxylate groups of the high density CS side chains of aggrecan provides a high fixed charge density that is responsible for the imbibition of water and the swelling pressure of the NP that provides hydrodynamic weight bearing properties to the composite disc structure.^40^, ^41^, ^42^

The NP is enclosed by the annulus fibrosus (AF), a tissue rich in interconnected lamellar sheets of fibrillar type I and II collagen. The type II collagen‐rich hyaline cartilage of the cartilaginous endplates (CEPs) interface with and firmly attach the IVD to the bone of the vertebral bodies (VBs).^43^ Type I collagen has the highest concentration in the outermost annular lamellae, while type II collagen is most concentrated in the NP as a random network of collagen fibers that entrap aggrecan‐hyaluronan (HA) ternary macro‐aggregates stabilized by link protein.^39^, ^44^ Type I and II collagens form counter gradients in the IVD with type I collagen concentrated in the outer AF providing tensile strength, its content decreases towards the central NP while type II collagen is concentrated in the NP decreasing towards the outer AF.^45^, ^46^, ^47^, ^48^ Type XI collagen in hybrid collagen I fibers interact with the HS chains of perlecan in the PCM further stabilizing the disc cell PCM particularly in the fibrocartilaginous AF.^49^

**FIGURE 3:**
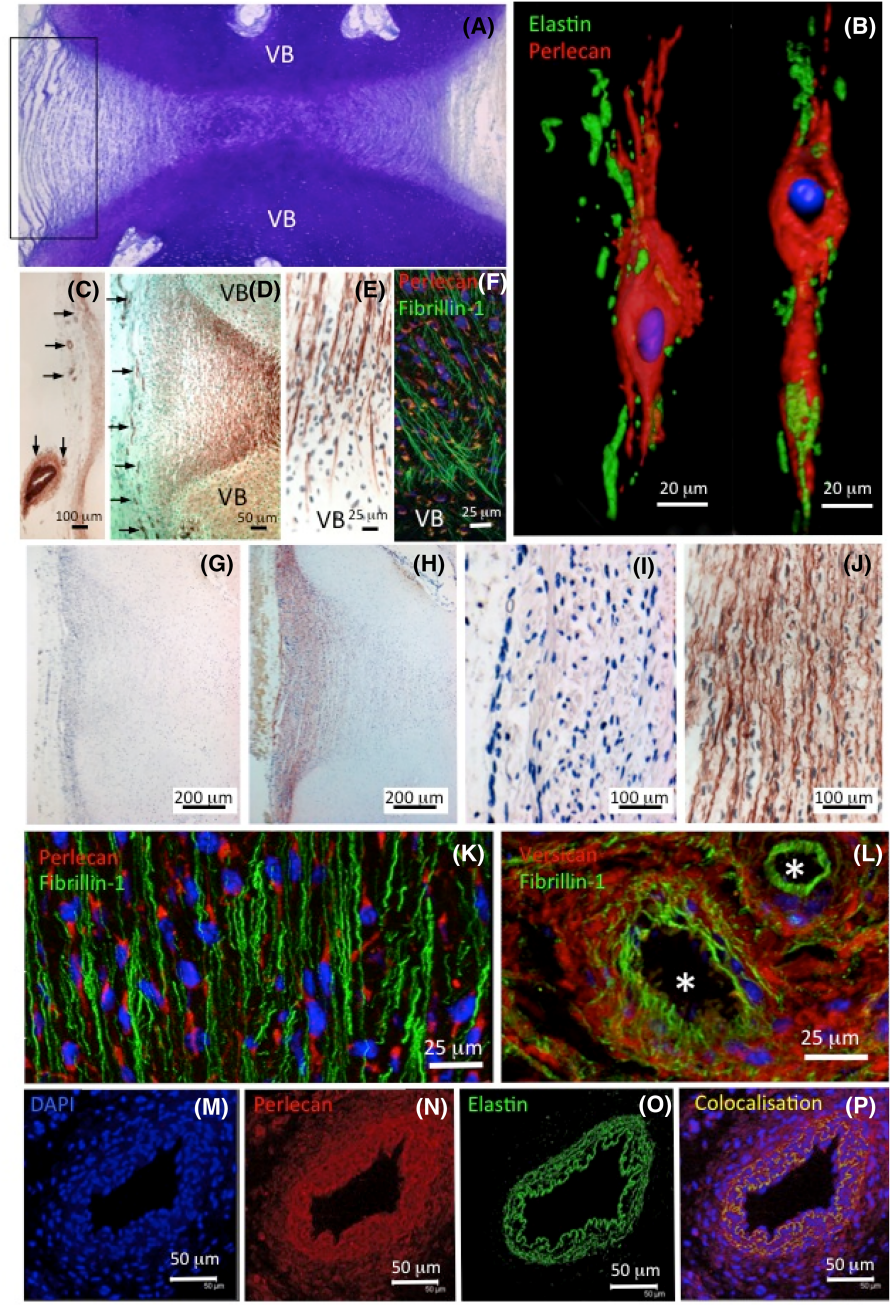
Structural organization of the intervertebral disc (IVD). Macroscopic toluidine blue stained fetal IVD and superior and inferior vertebral bodies (VBs) with the area of interest indicated by the boxed area in (A), the VBs are cartilaginous at this stage of spinal development. Demonstration of the production of elastin and fibrillin‐1 in close association with perlecan by ovine annulus fibrosus (AF) cells and colocalization of these components in outer annular blood vessels in a 14 weeks gestational age human fetal IVD. Surface rendered confocal images of two outer AF cells demonstrating fluorescent localization of elastin and perlecan (B). Small blood vessels (arrows) in the outer AF in an elastin‐stained section (C) and in a perlecan‐stained section (D). Elastin (E) and fibrillin‐1 fibers (F) anchor the IVD to the VB. Low power bright‐field images depict elastin fibers in the outer AF: Negative control (G) elastin immunolocalization (H). Higher power images of elastin immunolocalizations in outer AF: Negative control (I), elastin immunolocalization (J). Confocal images demonstrating fibrillin‐1 fibrils in the outer AF and localized around outer AF blood vessels (*). Versican is also found in the AF and localizes with elastic components in the AF (L). Confocal images of an outer AF blood vessel showing DAPI (M), perlecan (N), elastin (O), and perlecan‐elastin colocalizations (P). In confocal images, Z‐stacks of optical sections (F–I) were taken through the full thickness of tissue sections at 0.4–0.6 μm increments and maximum intensity type reconstructions prepared from image stacks using Leica Confocal Software. Areas of colocalization of perlecan and elastin were evident as yellow stained regions (I). Perlecan and elastin fluorescent localizations were conducted with red (Alexa 594) or green (Alexa488) fluorochromes. Nova red was the chromogen used in bright‐field images. Primary mouse anti‐bovine α‐elastin (MAb BA4) and rat monoclonal anti‐perlecan domain‐IV (mAbA7L6), and a MAb raised to the Pro rich region of fibrillin supplied by Prof Penny Handford, University of Manchester were used for the localizations. Images reproduced from reference 53 and images (B–E) reproduced from references 20, 54 with permission.

AF cells also synthesize elastin in close association with perlecan (Figure 3A–E). Elastin is layed down as interconnecting strands between adjacent lamellar layers, providing flexibility to this collagen rich tissue.^50^, ^51^, ^52^ Collagen networks are essentially inextensive structures and elastin provides important elastic recoil properties to the AF.^52^ Elastin also colocalizes with perlecan in small blood vessels in the outer AF of the fetal IVD and with paraspinal blood vessels^53^ (Figure 3G–I). Fibrillin microfibrils are also found associated with these elastic fiber networks in the AF and these contribute to their elastic properties.^20^, ^50^, ^51^, ^52^, ^54^, ^55^ Elastin and fibrillin‐1 fibrils anchor the IVD to the superior and inferior VBs.

**FIGURE 4:**
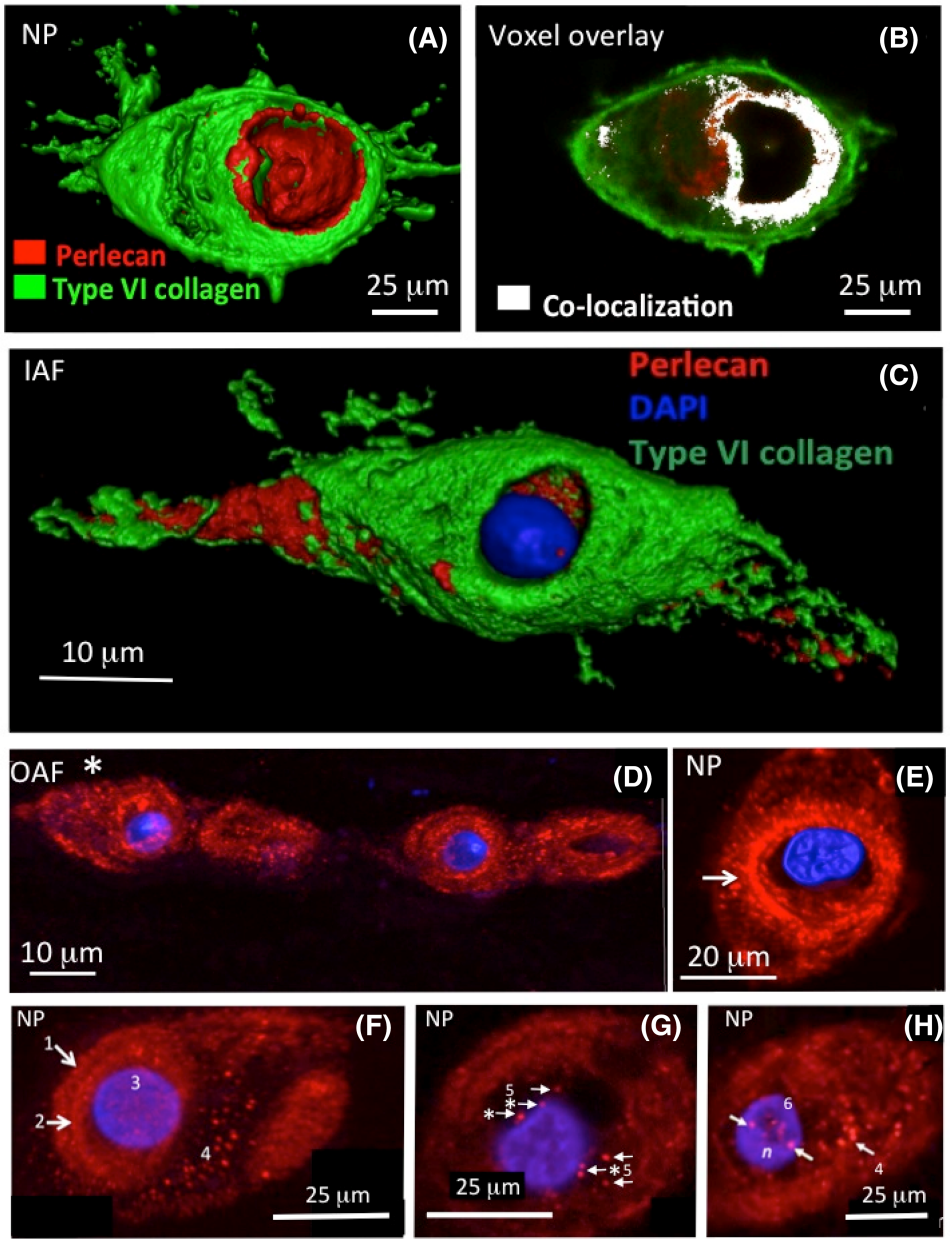
Perlecan is produced by annulus fibrosus (AF) and nucleus pulposus (NP) cells and forms part of a chondron structure along with type VI collagen which surrounds the cells. In image (A), the cell has fallen out of the chondron structure during histological processing, leaving perlecan attached to the surrounding type VI collagen of the chondron. Fluorescent perlecan‐type VI collagen 3D surface rendered confocal images of an NP and AF cell (A, C), voxel overlay perlecan and type VI collagen co‐localization (white region) of an NP cell (B) in ovine NP and AF cells. Perlecan localization was conducted with rat monoclonal anti‐perlecan domain‐IV (mAbA7L6) antibody and red (Alexa 594) fluorochrome labeled secondary antibody. Type VI collagen was immunolocalized using rabbit polyclonal anti‐type VI collagen antisera (VIb), a gift from Dr Shirley Ayad, Manchester University^75^ and Alexa 488‐conjugated goat anti‐rabbit secondary antibody. Nuclei were stained with DAPI 4′,6‐diamidino‐2‐phenylindole. Confocal image Z‐stacks of optical sections (D–H) were taken through entire tissue sections at 0.4–0.6 μm increments and maximum intensity type reconstructions were prepared from image stacks using Leica Confocal Software. Labeled features in images (F–H) are: (1) Outer edge of chondron; (2) Pericellular matrix; (3) NP; (4) Punctate deposits of perlecan in chondron; (5) Perinuclear punctate deposits of perlecan; (6) Intranuclear perlecan deposits. Images reproduced from reference 65 with permission.

Individual IVD cells are surrounded by an extensive ECM and a protective PCM, which together with the cells forms a “chondron.” This PCM is rich in type VI collagen and perlecan, which facilitates cell‐ECM communication (Figure 4).^56^, ^57^ Similar to its role in articular cartilage^3^ and other fibrocartilaginous tissues such as the meniscus,^3^, ^56^, ^58^, ^59^, ^60^, ^61^, ^62^, ^63^, ^64^ perlecan in the chondron surrounding the disc cell acts as a cytoprotectant molecule, preventing the collagen type VI network from mechanically overloading IVD cells.^65^ Perlecan also sequesters growth factors which act as a convenient reservoir for the nutritionally deprived disc cell and also provides disc cells with mechanosensory properties. The PCM has critical roles to play in the mechanobiology of cells in weight bearing tissues like the IVD.^64^

### 1.4 Structural aspects of the IVD that contribute to its functional properties

The IVD is a composite structure which provides strength and flexibility, as already discussed. Pericellular type VI and XI collagen and perlecan distributed in chondron‐like structures,^24^, ^49^ have important mechanotransductive roles facilitating communication between IVD cells and their biomechanical micro‐environment (Figure 1). This equips the disc cell with the ability to perceive micromechanical changes in the ECM,^3^ allowing the cell to orchestrate compensatory or homeostatic changes in tissue composition and organization.^31^, ^66^ Perlecan, localized in the collagen VI/XI rich chondron‐structure surrounding IVD cells, provides them with the ability to regulate the biosynthetic response of NP cells to osmotic loading to regulate chondrogenesis.^31^ Perlecan also interacts with fibrillin‐1 and elastin and this contributes to the viscoelastic properties of IVD tissues.^20^, ^24^, ^53^, ^54^ As already noted, pericellular perlecan has roles in cell‐ECM communication in weight‐ and tension‐bearing connective tissues such as the IVD.^3^

### 1.5 Nuclear perlecan

Perlecan has also been localized in the nucleus of disc cells,^67^ where it may have direct gene regulatory properties, but this still has to be determined. A number of other NP heparan sulphate proteoglycans (HSPGs) have also been identified^67^ and their functional properties in the NP also remain to be established.^67^ The resident IVD cell populations in the NP exist as single round cells surrounded by an abundant ECM, cells in the AF exist as strings of elongated fibroblastic cells between collagenous lamellae.^68^, ^69^, ^70^

### 1.6 Cytoprotection and nutrition

The IVD is one of the largest avascular and aneural tissues in the human body. IVD cells have adapted to survive in a hostile weight bearing, low oxygen tension, low pH environment with poor nutrition.^71^ The scant nutrition disc cells receive is by diffusion from the capillary networks in the VBs underlying the CEPs.^72^ With aging, structural changes in the CEPs and VB's can compromise this nutritional route to the resident IVD cell populations placing them in a precarious situation effecting their viability^73^, ^74^ and ultimately leading to cell death particularly in the NP, the region most distant from the VB capillary networks. Perlecan sequesters growth factors which are prone to enzymatic degradation and protects these increasing their biological half‐life. Perlecan in the PCM of IVD cells is thus conveniently located to supply these growth factors to IVD cells and may promote cellular survival.

### 1.7 Degradation of the IVD

**FIGURE 5:**
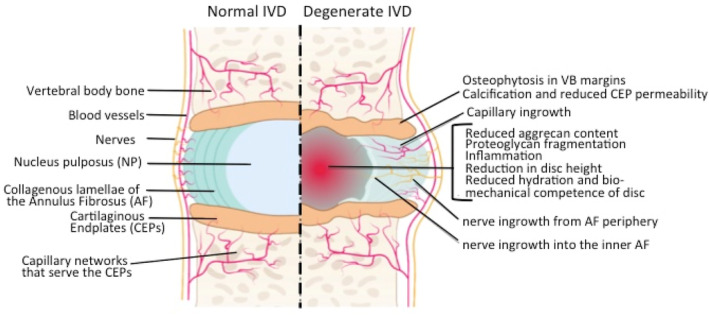
Schematic depiction of an intervertebral disc (IVD) showing features that characterize normal IVDs and changes in structural organization of degenerate IVDs. Figure modified from reference 78 with permission.

Mechanical destabilization of the IVD using a controlled outer annular incision has been used experimentally to induce IVDD.^76^ Mechano destabilization results in disc cells releasing matrix metalloproteases (MMPs) and inflammatory mediators.^77^ This leads to a hostile environment in the IVD where degradation of PGs such as aggrecan and perlecan occurs in the NP compromising IVD's role as a viscoelastic cushion that provides weight bearing properties to the composite disc structure (Figure 5).

**FIGURE 6:**
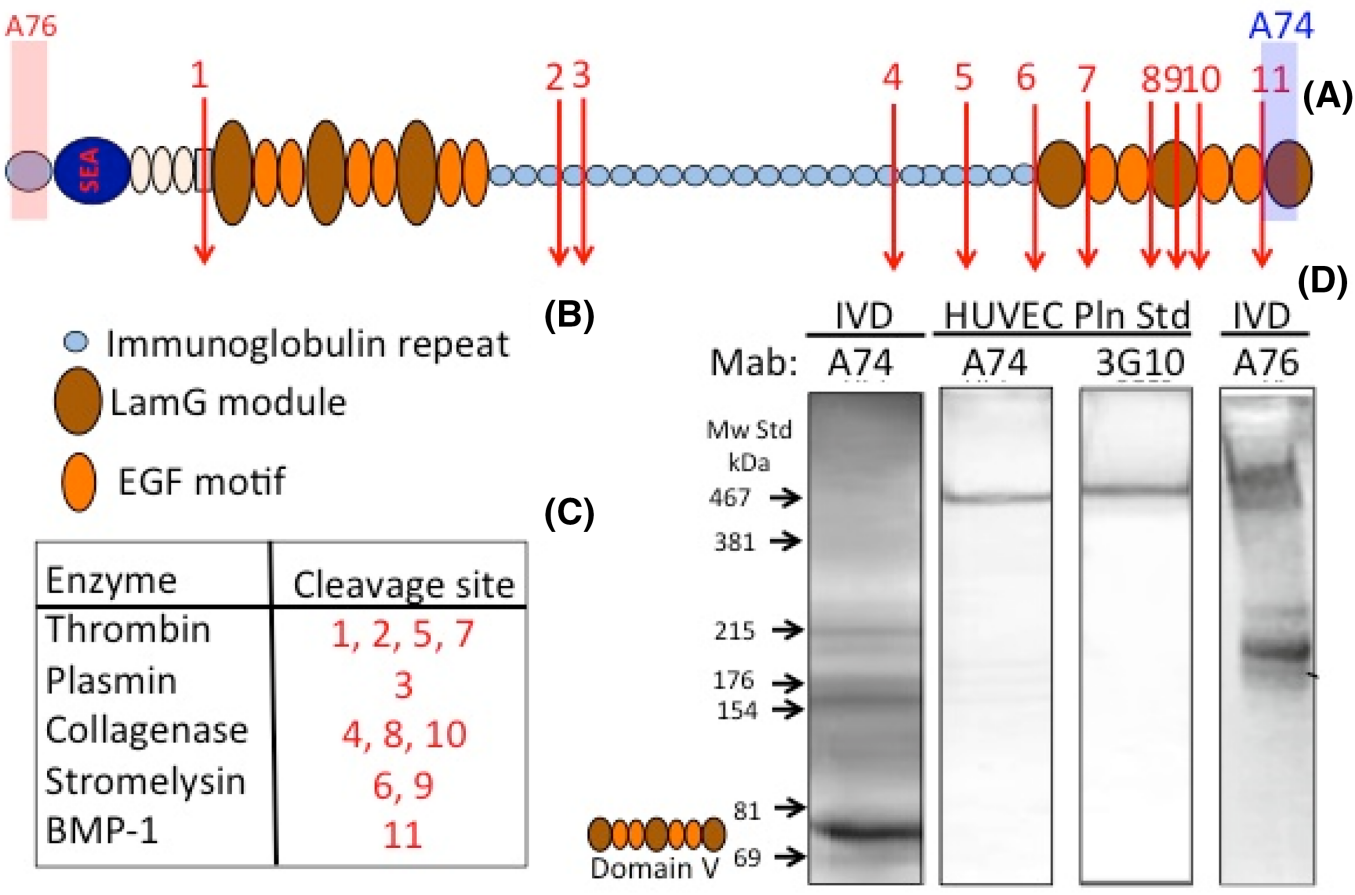
The modular structure of perlecan showing the glycosaminoglycan (GAG) substituted domain I unique to perlecan. Laminin A motifs and low density lipoprotein receptor like domain of domain II, the laminin G and epidermal growth factor motifs of domain III and V and multiple immunoglobulin repeats of domain IV (A). Key to major core protein modules (B). Protease cleavage sites in perlecan core protein (C). Western blot showing an 80 kDa perlecan domain V fragment detected using MAb A74. Figure modified from reference 12. HUVEC, human umbilical vein endothelial cell.

Intact aggrecan in the normal IVD has water imbibing properties due to its high density of glycosaminoglycan (GAG) side chains and counter‐ion content.^40^, ^41^, ^42^ Imbibition of water in the NP provides an internal hydrostatic pressure in this tissue through the Donnan effect and this provides the IVD with its weight bearing properties. Osmolarity also regulates gene expression in IVD cells as part of the mechanobiologic response to loading.^79^ Degradation of aggrecan during IVD degeneration severely disrupts the normal metabolism of disc cells and eventually results in dehydration of the NP, a reduction in disc height and a severe reduction in the biomechanical competence of the IVD.^44^, ^80^ Perlecan is highly susceptible to degradation by MMPs and a number of other proteases also degrade perlecan in domain IV and V (Figure 6A–C). This also results in the release of C‐terminal perlecan fragments, including domain V and LG1–LG2, and LG3 fragments of domain V (Figure 6D).^12^ Perlecan can be degraded by plasmin, the BMP family of tolloid proteases,^81^ and a range of MMPs.^12^, ^82^ Domain IV of perlecan is extensively degraded by MMP‐7 in prostate cancer.^83^, ^84^ Perlecan domain‐V has been proposed as a functional PG in its own right with roles in the stabilization of the blood brain barrier (BBB) and in tissue fibrosis.^85^ Perlecan domain‐V has therapeutic properties after experimental ischemic stroke and promotes neurogenic brain repair.^86^, ^87^ Perlecan may also be a useful treatment for vascular dementia^88^ thus it has been proposed as a therapeutic agent in tissue recovery in post‐ischemic stroke in humans.^89^, ^90^, ^91^ Recombinantly expressed human perlecan domain V is a bioactive molecule that actively promotes angiogenesis and vascularization of implanted biomaterials eliciting a strong repair response. Proteolytically released PG fragments have been termed matricryptins, reflecting the hidden biological properties of these domains,^92^ some matricryptins elicit a repair response in damaged tissues.^93^ The released matricryptic fragments of perlecan have potential roles in matrix repair processes and have been proposed to also regulate angiogenic processes limiting tumor growth.^21^ Upon degradation of aggrecan and an impairment in the weight bearing properties of the IVD, secondary compensatory fibrotic changes in the IVD occur further compromising the IVD's viscoelastic properties and it becomes less compliant and resilient and more brittle and susceptible to the development of radial and circumferential tears and separation of adjacent annular lamellar layers (de‐lammelation) upon biomechanical overload. Accumulated fatigue stresses can also lead to fracture of Sharpey fiber AF anchorage points of the IVD to the VB leading to formation of rim lesions. When these defects communicate with the outer margins of the AF clefts develop and an ingrowth of blood vessels and nerves can occur into the degenerate IVD when it becomes depleted of its excluding space‐filling aggrecan. An influx of inflammatory cells occurs along these clefts and an inflammatory environment is generated in the degenerate IVD conducive to the development of nociceptive nerves and mechanoreceptors.^94^ This contributes to the perception of low back pain (LBP) when the biomechanically incompetent degenerate IVD no longer dissipates spinal forces adequately.^94^ Elevated levels of mechanoreceptors in the degenerate IVD makes these sensitive to overloading and along with nociceptive nerves contributes to the perception of LBP. Pain generation becomes accentuated in the lower lumbar regions due to axial biomechanical forces no longer being adequately redistributed/dissipated with these forces being transmitted down the spine and are concentrated in the lower lumbar spinal levels.^94^ In the erect human spine the juncture of the flexible lumbar region with the immobile pelvic lumbosacral spine is a major determinant of the PG metabolism of the spine and is a symptomatic spinal region with a high incidence of IVDD and LBP.^95^ With IVDD component PGs become degraded into fragments, aggrecanase and MMP neoepitope antibodies have demonstrated the disassembly of aggrecan‐HA‐link stabilized macroaggregates that leads to a lowering of water imbibition and a reduction in disc height in IVDD and a loss of biomechanical competence. Perlecan is also susceptible to degradation by a range of MMPs and serine proteases and heparanase^82^ (Figure 6).

### 1.8 Generation of bioactive matricryptic proteoglycan fragments

A matricryptin is a module within a PG core protein which has a hidden biological activity. When the matricryptin is released from the PG core protein by proteolytic processing a new biological activity may become evident. Some of these PG fragments termed matricryptins or matrikines have interesting biological properties of potential application in repair biology or as anticancer agents.^92^, ^93^ The small leucine rich proteoglycans (SLRPs) are degraded into a number of fragments with IVDD.^96^, ^97^, ^98^, ^99^, ^100^, ^101^ Lumican contains peptide modules that act as MMP inhibitors, lumcorin is a peptide derived from lumicans leucine rich repeat (LRR) domain 9 which displays MMP inhibitory activity.^101^ A peptide designed from the 13 C‐terminal amino acids of lumican (LumC13) binds to anaplastic lymphoma kinase (ALK)5/transforming growth factor (TGF)BR1 (type 1 receptor of TGFβ) and promotes wound healing.^97^ Lumican derived peptides also inhibit melanoma spread.^100^ Perlecan is also susceptible to enzymatic degradation particularly in domain IV and V.^12^ An 80 kDa C‐terminal fragment of perlecan is a prominent component of degenerate IVDs (Figure 6D). Perlecan domain V promotes repair of the BBB following ischemic stroke.^89^, ^91^, ^102^, ^103^ Perlecan domain V promotes laying down of an endothelium and has useful traits that promote incorporation of vascular grafts and improves the performance of coated implants in tissue repair.^18^, ^104^, ^105^ The 80 kDa C‐terminal fragment of perlecan in degenerate IVDs may promote ingrowth of blood vessels into the degenerate IVD^106^ and in chondroid metaplasia in degenerate IVDs.^68^

### 1.9 Perlecan has prominent roles in early rudiment cartilage development

**FIGURE 7:**
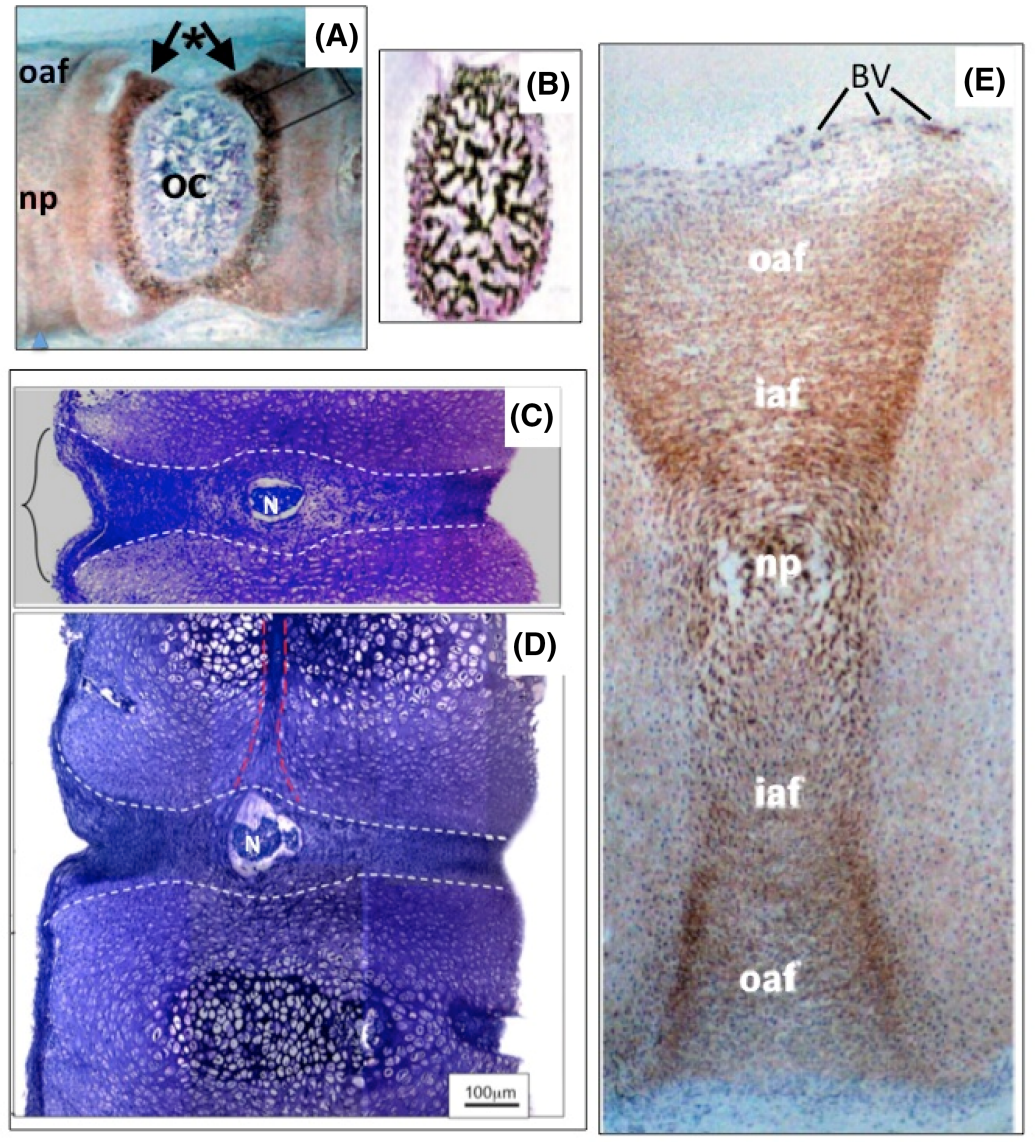
Perlecan as an early chondrogenesis marker but which also participates in osteogenic development of the spine. Immunolocalization of perlecan in hypertrophic chondrocytes surrounding an ossification center at 14 weeks gestational age in a human fetal spinal rudiment (A). Von Kossa staining confirms mineralization in the ossification center (B). The spinal rudiment is cartilaginous at 12 weeks gestational age (C, D) and remnants of the notochord (N) are still evident in these specimens. Hypertrophic chondroid cell masses are evident in what will become the ossification center in the vertebral body rudiment. The developing intervertebral disc (IVD) is indicated by dotted lines. Perlecan expression is delineates the extent of the developing IVD (E). Images modified from reference 35 with permission. BV, blood vessels; iaf, inner AF; np, nucleus pulposus; Oaf, outer AF; OC, ossification center.

Perlecan is an early marker of chondrogenesis^63^ and is widely expressed during the development of rudiment cartilages during skeletal development^107^ and chondro‐osseus development of the human fetal spine^35^ (Figure 7). Perlecan promotes chondrocyte proliferation, differentiation and matrix stabilization.^25^ The hypertrophic chondrocytes which establish ossification centers during fetal human spinal development have similar morphologies to chondroid cellular arrangements in mature connective tissues. Chondroid metaplasia may thus represent a recapitulation of the early rudiment cartilage development seen in skeletogenesis. Perlecan has prominent roles to play in these morphogenetic processes. Over‐expression of perlecan in chondroid cell arrangements in OA cartilage may represent an attempted repair response.^108^ Furthermore, perlecan prominently delineates small stem cell niches in human fetal knee and hip cartilages which have roles in the development of diarthrodial joint development (Figure 2A).^2^ Further studies have established that chondroprogenitor stem cells express CS sulphation motifs such as 3B3[−] and 7D4^109^ and hypertrophic chondrocytes in chondroid cell masses in the human fetal elbow^110^ and associated with annular lesions in an experimental model of IVD degeneration,^68^ these are considered to be markers of tissue morphogenesis.^109^ Furthermore, 3B3[−] and 7D4 cell surface CS‐sulphation neoepitope markers have been used to isolate chondroprogenitor stem cells from cartilage.^111^, ^112^ The isolated stem cells have been shown to be capable of synthesizing full depth neo‐cartilage in vitro with spatial and structural organization of collagens and PGs equivalent to that found in native articular cartilage.^111^ These CS sulphation epitopes are expressed in normal fetal human and young bovine cartilage by resident stem cells,^29^ and in neonatal articular and growth plate cartilage.^113^ HS on perlecan has prominent roles in cartilage development and tissue morphogenesis^21^, ^28^ and considerable potential in tissue repair biology^22^ and cartilage repair and regeneration.^26^, ^27^

### 1.10 Chondroid metaplasia as a partial repair response to IVDD

**FIGURE 8:**
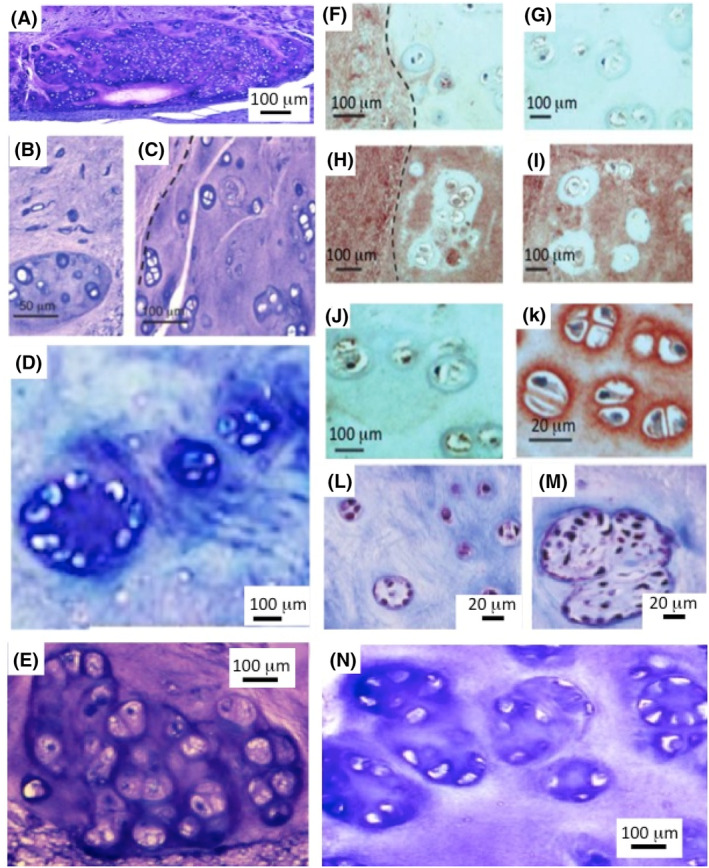
Histological demonstration of a chondroid cell nest in the nucleus pulposus (NP) of a normal 2‐year‐old ovine intervertebral disc (IVD) (A). These chondroid cell nests occur within a toluidine blue rich basophilic matrix separate from the surrounding NP. Chondroid cells are significantly larger than NP cells and have a rounded morphology. Some chondroid cells are dividing in the cell nest but there is no evidence of cell division in the surrounding NP cells (B, C). Cell clusters observed in the vicinity of an annular lesion in the inner annulus fibrosus (AF) depleted of extracellular matrix (ECM) proteoglycan (D). A chondroid cell nest in the NP (E). Immunolocalization of versican in the matrix surrounding a chondroid cell nest (F) and within the nest shows the surrounding NP is positive for versican but the chondroid cell nest is negative (G). Aggrecan immunolocalizes throughout the surrounding NP (H) and within the chondroid cell nest but not in the pericellular matrix surrounding chondroid cells (I). Hyaluronidase‐treated control slide (J). Hyaluronan was localized in this pericellular region using a biotin aggrecan G1 bioprobe detected using an avidin horseradish peroxidase (HRP) secondary reagent (K). Primary antibodies to aggrecan and versican and HA bioprobe were as in reference 120, chromogen used was NovaRed. Chondroid cells occur in degenerate grade III (L) and IV human IVDs (M). Chondroid cell clusters in a beagle IVD (N). Images reproduced from reference 120 with permission. Images (L and M) provided by Prof. HE Gruber, and Dr. EN Hanley Carolinas Medical Centre, Charlotte, USA.

Nests of chondroid‐like cells have been observed in basophilic cell nests in normal ovine NPs (Figure 8A–D). Small cell clusters of similar morphology have also been observed adjacent to annular lesions in an ovine model of IVDD (Figure 8E).^114^ Chondroid cell nests have been observed in the normal ovine IVD (Figure 8A). These cells have a dissimilar morphology to resident NP cells, and are significantly larger (Figure 8B, C). Cells in these chondroid cell nests undergo cell division which is rarely seen in the resident NP cell populations.

### 1.11 Expression of PGs by chondroid‐like cells

Perlecan protein and mRNA is significantly up regulated in these chondroid‐like clonal cellular arrangements.^108^ This has been proposed to be an attempt to stabilize the cartilage ECM but is an incomplete repair response.^108^, ^115^ Immunolocalization of aggrecan in ovine NP tissues showed a widespread distribution throughout the NP including chondroid cell nests except in the PCM of chondroid cells (Figure 8H). Versican was localized in the NP but not in these chondroid cell nests. HA was localized in the PCM of chondroid cells in regions where aggrecan was excluded but was not a feature elsewhere in the NP (Figure 8F–K).

**FIGURE 9:**
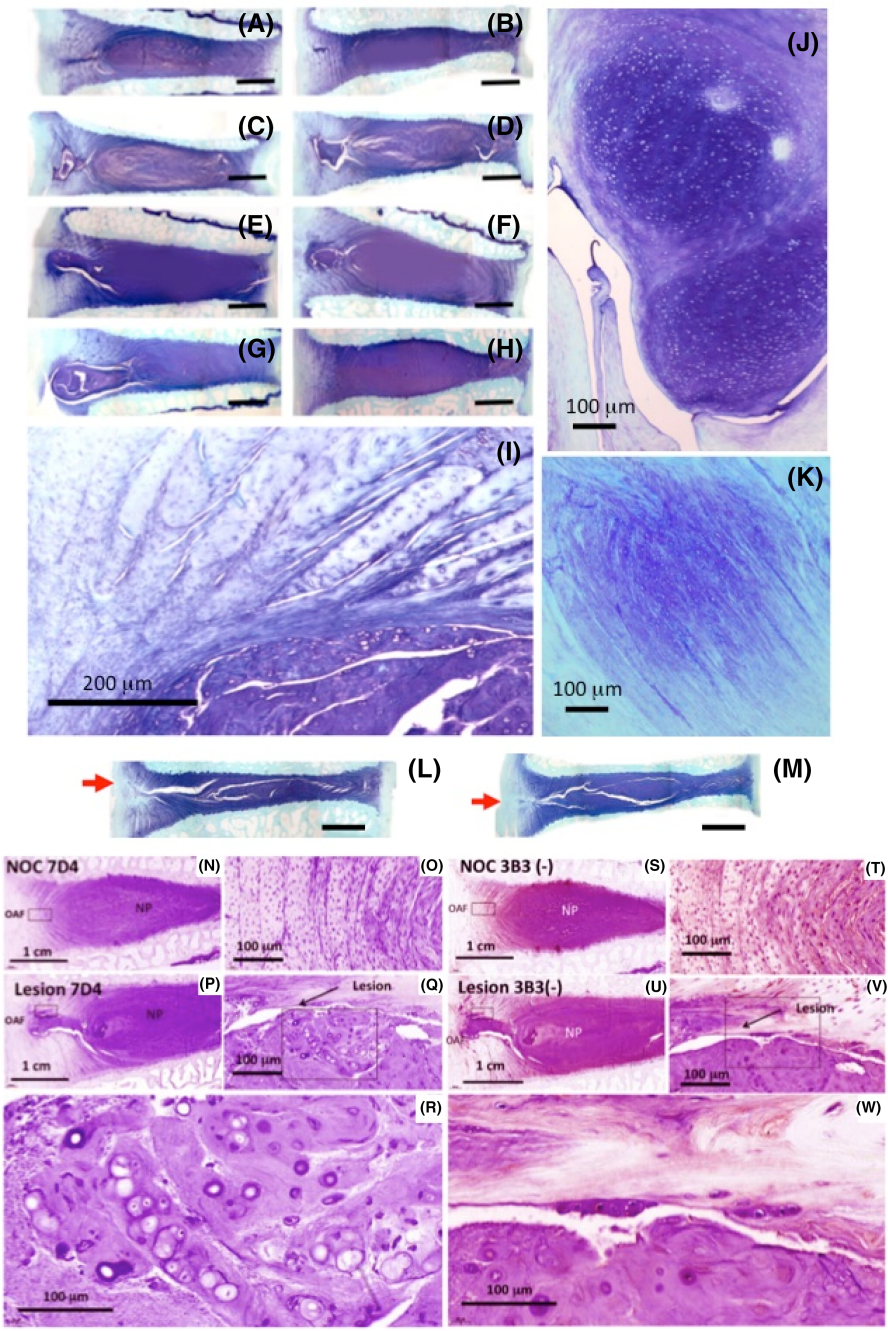
Healing in lesion‐affected ovine intervertebral discs (IVDs) in response to exogenously applied bone marrow stromal stem cells (A, B). Chondroid deposits in the annulus fibrosus (AF) around the annular lesion in lesion‐affected IVDs that did not receive exogenous stem cells (C, D). This chondroid deposition of tissue apparently arising from the nucleus pulposus (NP) was more prominent in ~10% of all lesion affected IVDs that received stem cells (E–G). A normal nonoperated control disc is shown for comparison (H). Small rounded cells in the chondroid cell mass had a morphology dissimilar to other IVD cells (I). In some cases, the chondroid outgrowth from the NP merged with the AF lamellae (J). Chondroid cell masses have also been observed in an ovine tendinopathy model induced by extracellular matrix (ECM) destabilization after a surgical incision (K), In all cases where chondroid cell masses were observed, the annular lesion failed to propagate deeply into the IVD through to the contralateral AF. Extensive AF lesions in this model where no chondroid deposits were evident disrupted the internal structure and resulted in a reduced disc height (L, M). Images (A–J, L, M) reproduced from reference 114. Image (K) supplied by Dr. MM Smith, University of Sydney. Chondroid cell masses in lesion affected IVDs express CS‐sulphation motifs 7D4 and 3B3[−] which are stem cells of tissue morphogenesis. Nonoperated discs showing expression of 7D4 and 3B3[−] CS sulphation motifs (N, O, S, T). Lesion affected IVDs (P–R; U–W) boxed areas are shown at higher magnification (Q, V). Chondroid cell masses in lesion affected IVDs show 7D4 and 3B3[−] expression with the boxed area shown at higher magnification (R, W). Hypertrophic chondroid like cells express these CS sulphation motifs. NOC, nonoperated control. Images reproduced from reference 68 with permission. Scale bars in (A–H, L, M) 10 μm.

Chondroid metaplasia has been observed in an ovine annular lesion destabilization model of IVDD,^68^, ^114^ focused in regions of the inner AF adjacent to the NP (Figure 9). Higher power images showed the small rounded cells of the chondroid cell mass and in some cases healing of the inner lesion with the AF showing continuity with the NP, however the normal annular architecture was disturbed but propagation of the AF lesion into the inner AF was prevented. Chondroid cell masses have also been observed in an ovine tendinosis model induced by a surgical incision which induces mechanical destabilization (Figure 9K). Perlecan expression is also very significantly elevated in this tendinosis model.^116^ Chondroid metaplasia also occurs in chondrodystrophic canine IVDs and in non‐chondrodystrophic canine breeds but to a lesser extent (Figure 8N).^117^, ^118^ Chondroid metaplasia has also been observed in equine IVD degeneration^119^ and in an ovine experimental model of IVD degeneration.^76^, ^114^

Chondroid cell masses have been reported in grade 3 and 4 degenerate human IVDs (Figure 8I,M)^120^ and in a surgically induced mechanical destabilization tendinosis model.^121^, ^122^ A few cases of chondroid metaplasia in cases of fibrocartilaginous dysplasia (fibrous dysplasia with massive cartilaginous differentiation) of bone have been reported in the femur and tibia.^123^, ^124^, ^125^, ^126^ Histologically, fibrocartilaginous dysplasia is characterized by islands of hyaline cartilage in fibro‐osseous lesions, and these cell masses resemble the chondroid deposits seen in other tissues.

### 1.12 Stem cell marker expression in chondroid cell clusters in human IVDs

Immunolocalizations of degenerate human IVDs has identified chondroid cell clusters^120^, ^127^, ^128^ that express progenitor stem cell markers (7D4, 4C3, 6C3, 3B3[−]),^127^ flow cytometry of these cultured cells showed they expressed CD73, CD90, and CD105 stem cell markers and had similar profiles to bone marrow‐derived mesenchymal stem cells.^128^ Small groups of chondroid cells which express 7D4 and 3B3[−] stem cell markers have also been observed in an ovine annular lesion model of experimental disc degeneration in the vicinity of annular lesions.^68^, ^76^, ^114^, ^129^ These CS sulphation epitopes are expressed in normal human fetal and bovine cartilage by resident stem cells.^29^ CS sulphation motifs 3B3[−] and 7D4 are focally expressed in chondroid cell masses in the ovine destabilization model of IVDD in the inner AF^68^, ^114^ These CS sulphation motifs have previously been shown to be expressed by mesenchymal stromal stem cells involved in tissue morphogenesis.^109^ The chondroid cells in these destabilized regions of the inner AF thus appear involved in a tissue stabilization process where the inner lesion integrates with surrounding tissue however a reattainment of normal prestressed AF lamellar tissue architecture does not occur but the lesion is prevented from further propagation into internal regions of the IVD.

### 1.13 Stem cells are mechanoresponsive cell types

Resident and administered stem cells are mechanoresponsive and guided by in‐situ mechanical loading in their responses in tissue repair processes.^130^, ^131^, ^132^ Tissue architecture and the local biological environment also influence stem cell behavior in defect sites.^133^

Perlecan has key roles to play in mechanotransductive processes that guide resident connective tissue cell populations and progenitor stem cells, whether they be resident in the tissues or exogenously administered. Chondroid deposits in the ovine experimental model of disc degeneration were focused on the inner lesion adjacent to the NP and although this did not result in reattainment of normal annular structure it did prevent further propagation of the lesion into the IVD. Discrete chondroid cell nests have been observed in the ovine and human IVD (Figure 8D,I,M) and these may contain stem cells that become activated when an annular lesion perturbs normal biomechanical forces experienced by the NP, possibly resulting from growth of chondroid tissue from the NP and its participation in tissue repair responses.^120^, ^134^, ^135^, ^136^ Similar cell nests in OA cartilage adjacent to surface fibrillations have been shown to express elevated perlecan levels.^108^ Disruptions in the normal collagenous fibrillar organization in tissue defect sites have been suggested to guide stem cells to the defect site where they can promote repair processes.^137^ Resident fibrocartilage stem cells have been used to regenerate and repair cartilage showing the potential of this approach in the repair of weight bearing and tension resisting connective tissues.^138^ Furthermore, intra‐articular treatment with the Wnt inhibitor sclerostin maintained the fibrocartilage stem cell pool promoting regeneration of cartilage in a temporomandibular joint injury model demonstrating the adaptability of resident stem cells in such tissue repair and regenerative processes.^138^, ^139^

### 1.14 IVD stem cells

Like all other musculoskeletal tissues, the IVD contains a progenitor stem cell population with roles in its development and these cells would also be expected to participate in tissue repair processes.^120^, ^140^ In human fetal cartilages these stem cells are found in niches surrounded by perlecan which has roles in maintaining the viability of a recycling quiescent stem cell population and a sub‐population of stem cells which differentiate to a pluripotent phenotype and develop migratory properties that allows these cells to escape the niche environment to home to sites of IVD damage where they can participate in a tissue repair response.^2^, ^110^, ^141^, ^142^, ^143^, ^144^, ^145^, ^146^, ^147^ We believe that chondroid cell masses found in degenerate IVDs and described in the present study represent this migratory stem cell population. Isolation and characterization of these cell masses from the IVD^128^ has shown that they express progenitor or notochordal cell markers (chondroitin sulphate epitopes [3B3(−), 7D4, 4C3, and 6C3], Notch‐1, cytokeratin 8 and 19) using immunohistochemical examination and stem cell markers assessed by flow cytometry (CD73, CD90, and CD105 positivity). Thus these chondroid cell masses are similar to bone marrow‐derived mesenchymal stem cells. Small groups of chondroid cells which express 7D4 and 3B3[−] chondroitin sulphate stem cell markers have also been observed in an ovine annular lesion model of experimental disc degeneration in the vicinity of annular lesions.^68^ Mesenchymal stromal stem cells show a tremendous potential for the repair of damaged IVD tissues.^78^, ^148^, ^149^, ^150^, ^151^, ^152^, ^153^ When exogenously administered to degenerate IVDs in an ovine model of experimental IVDD, mesenchymal stromal stem cells successfully repaired a large 6 mm × 20 mm annular defect and resulted in the reattainment of a normal IVD composition and recovery of normal IVD biomechanics.^114^, ^129^ This is a large defect and its repair was a significant finding firmly establishing the efficacy of stem cells for tissue repair. We believe that the cell stimulatory and tissue reparative properties of perlecan^11^, ^21^, ^26^, ^27^, ^154^, ^155^ also significantly contributed to the successful repair of IVD tissues. Perlecan has previously been shown to participate in the repair of the BBB following ischemic stroke^87^, ^89^, ^102^ and spinal cord basement membranes following traumatic injury.^156^ The chondroid cell masses which we have observed in degenerate IVDs are further evidence of an endogenous resident stem cell population present as cell clusters within the IVD.^120^

### 1.15 The role of the PCM and mechanosensitive ion channels in mechano‐osmotic signaling in IVD mechanobiology

Similar to articular cartilage^157^ and meniscus,^158^ cells of the AF and NP respond to a variety of physical signals that are secondary to mechanical loading of the IVD complex.^79^ Loading of the IVD due to activities of daily living will cause a complex and site‐specific array of mechanical, electrical, and osmotic signals in tissue, which will depend on loading type, magnitude, duration, and anatomic site of cell origin. While the exact mechanisms by which IVD cells respond to different physiologic or pathologic mechanical stimuli remain to be determined, it is now clear that the PCM plays a criticial role in mechanotransduction in the IVD^159^ As in articular cartilage, the PCM in the IVD appears to modulate the transduction of mechanical compression into osmotic changes in the pericellular environment, secondary to exudation of insterstitial fluid and increases in fixed charge density by compaction of proteoglycan.

IVD cells possess mechano‐osmotically‐sensitive ion channels such as transient receptor potential vanilloid 4 (TRPV4), a cation channel that serves to convert extracellular osmolarity into an intracellular biologic signal.^160^ Trpv4 is expressed in the NP, inner AF, cartilage endplate and vertebral growth plate in mouse IVDs.^161^ The TRPV4‐specific agonist GSK1016790A and antagonist GSK2193874 have been used to assess the functional response of AF cells to mechanical stimulation and quantified by gene expression profiling. In IVD cell culture, inhibiting TRPV4 reduces the hypo‐osmotic‐mediated production of IL‐1β and IL‐6, as well as the high‐magnitude strain‐mediated expression of IL‐6 and IL‐8.^162^ TRPV4 is expressed in the NP, inner AF, cartilage endplate and vertebral growth plate in mouse IVDs.^161^

TRPV4‐induced Ca2+ signaling is associated with Rho/Rho kinase (ROCK)‐dependent remodeling of the actin cytoskeleton and the formation of stress‐fibers.^161^ Cyclic‐tensile‐strain‐induced changes in Acan and Prg4 expression mediated by TRPV4 channel activation establish TRPV4 as a mechanosensor that regulates IVD mechanobiology.^163^, ^164^, ^165^ In IVD organ culture, activation of TRPV4 increased nuclear factor (NF)‐κB signaling and higher interleukin IL‐6 production, concomitant with the accumulation of GAGs and increased hydration in the NP that culminated in higher stiffness of the IVD.^163^ Sustained compressive loading of the IVD resulted in elevated NF‐κB activity, IL‐6 and vascular endothelial growth factor A (VEGFA) production, and degenerative changes to the ECM, whereas TRPV4 inhibition during loading mitigated the changes in inflammatory cytokines and protected against IVD degeneration.^166^ These results indicate that mechano‐osmotic signaling via TRPV4 plays an important role in both short‐ and long‐term adaptations of the IVD and could be targeted to prevent load‐induced IVD degeneration. A number of studies have demonstrated the responsiveness of AF and NP cells to short duration or long term dynamic compression which can result in a combination of anabolic and catabolic responses that may result in ECM remodeling or enhanced matrix synthesis. This experimental loading regime mimics the cyclical loading the IVD normally receives in day‐today activities and through which cell‐ECM feedback cues orchestrate IVD homeostasis.^167^, ^168^, ^169^ High mechanical strain applied in this manner induces deleterious secretion of inflammatory factors by disc cells, which contributes to degenerative changes in the IVD and to the generation of pain.^170^ An IVD organ culture system has been developed to investigate these pro‐inflammatory mediators and the degenerative features they induce in the IVD.^171^ A one strike loading organ culture model has also been developed to investigate the injurious effects of a single acute traumatic impact on the functional properties of the IVD.^172^ IVDD induced by a single high magnitude mechanical impact is not well understood. Using this model, a single hyperphysiological mechanical compressive impact on healthy IVDs resulted in a significant decrease in cell viability, an alteration in mRNA expression and an increase in ECM degradation. This model shows potential in the investigation of IVD changes in post‐traumatic degeneration and may identify novel biomarkers and therapeutic targets useful in prospective new treatment therapeutics.^172^

While the TRPV4 ion channel can regulate secretion of inflammatory mediators contributing to IVD degenerative changes and pain,^166^, ^173^ a recent study demonstrated that activation of the innate immune response through toll‐like receptors (TLRs) can also contribute to deleterious IVD changes in IVDD.^174^ Mechanically loaded rat IVDs displayed increased pro‐inflammatory mediators with static but not dynamic loading. This elevation in inflammatory cytokines was prevented by transforming growth factor‐*β*‐activated kinase (TAK)‐242, an inhibitor of TLRs.^175^, ^176^ This demonstrated that TLR4 had a direct role to play in the mediation of inflammatory responses in the IVD in response to injury induced by static loading.^174^

### 1.16 Up‐regulation of perlecan expression in specific tissue contexts may be a repair response

Upregulation of perlecan expression in fibrocartilaginous and cartilage lesions may represent a repair response and a recapitulation of fetal cartilage development giving credence to the use of perlecan in cartilage repair strategies. Deposition of a chondroid mass has been observed in tendon in a surgically induced mechanical destabilization tendinosis model,^121^ and perlecan expression is significantly up‐regulated in such lesions.^116^ Furthermore, Perlecan is an early chondrogenic marker in spinal development^35^ and in rudiment cartilage development^29^, ^63^ and has important roles to play in sensory regulation of disc cells and articular chondrocytes^3^ and shows potential in cartilage repair.^27^

A few cases of fibrocartilaginous dysplasia (fibrous dysplasia with massive cartilaginous differentiation) of bone have been reported in the femur and tibia.^123^, ^124^, ^125^, ^126^ Chondroid dysplasia has been observed in a model of experimental disc degeneration induced by an annular lesion.^68^ These cases may represent an attempted repair response and appear to result in stabilization of internal annular lesions although a return of normal annular structure does not occur but a localized cartilaginous repair tissue within the AF provides continuity between the NP and AF in a region previously destabilized by an annular lesion. These areas of chondroid tissue in the AF/NP interface appear very similar histologically to the nodular hyaline cartilage described in fibrous dysplasia in the femur and tibia.^123^, ^124^, ^125^, ^126^ Perlecan has roles in cartilage development and function and is an early marker of chondrogenesis.^21^, ^22^, ^63^

Perlecan is widely distributed in cartilage rudiments which also have a similar cellular distribution and matrix composition to nodular hyaline cartilage deposits. Perlecan promotes chondrogenesis in rudiment cartilage^22^ a transient scaffolding tissue that undergoes endochondral ossification to promote skeletal development.^107^ Interest has been shown in the use of perlecan for the repair of articular cartilage.^26^, ^27^, ^177^ Areas of chondroid dysplasia in experimental disc degeneration resemble rudiment cartilage morphology and composition apparently undergoing a repairative response.^68^ Perlecan may thus promote IVD repair giving some credibility to its use in repair of hyaline cartilage.^27^ Levels of perlecan protein and mRNA are up‐regulated in hypertrophic chondrocyte clusters in cartilage lesions in OA^110^ (Figure 4I–L). It is noteworthy that chondroid tissue in IVDs is also populated by hypertrophic cells that express 3B3[−] and 7D4 CS epitope markers of tissue morphogenesis and produced by chondroprogenitor stem cells responsible for cartilage development.^109^, ^110^

## 2 CONCLUDING COMMENTS

This study has reviewed perlecan's roles in IVD repair, spinal development, matrix stabilization and mechanosensory processes in weight bearing and tension resisting connective tissues. Chondroid cell masses identified in mechanically destabilized mature fibrocartilaginous connective tissues have a similar appearance to cellular arrangements in rudiment cartilage that undergo a chondroid metamorphosis in fetal spinal development. These chondroid masses in mature destabilized connective tissues may represent an attempted spontaneous repair response and a recapitulation of the developmental stages that occur during transformation of fetal rudiment cartilages into bone as part of the skeletogenesis process. Perlecan has important roles in rudiment cartilage development and is likely involved in these repair responses in mature tissues. The presence of perlecan Domain V in degenerate IVDs suggests it may participate in repair responses similar to its roles in the BBB following ischemic stroke and provides support to the prospective use of perlecan in cartilage repair strategies. A better understanding of perlecan's roles in tissue repair processes is essential before it can be prospectively harnessed to repair weight bearing and tension resisting connective tissues.

## 3 CONCLUSIONS

Chondroid metaplasia may be considered a compensatory response to altered mechanics that occur in connective tissues in specific contexts. Perlecan, as a regulator of mechanical and osmotic signaling in fibrocartilaginous and cartilaginous tissues, has properties applicable both to the early proliferative and mature matrix stabilization stages of this process. Furthermore, perlecan promotes the attainment of pluripotency and a migratory phenotype to progenitor stem cells consistent, with its localization around the periphery of stem cell niches in fetal rudiment cartilages. These stem cell populations have roles in normal tissue development. Nests of chondroid progenitor cells have been observed in the ovine IVD and in other tissue settings such as in fibrillated regions of OA cartilage. Clones of cells have been observed which overexpress perlecan suggesting its possible involvement in the chondroid metaplastic response we observed, such cell nests have also been observed in degenerate human IVDs. Chondroid metaplasia in the IVD is the replacement of fibrocartilaginous cells with chondrocyte‐like cells in response to mechanical destabilization, and has also been observed in paraspinal tissues in the degenerate spine.^178^ It is a benign condition found in connective tissues that have been exposed to chronic altered mechanical stress^179^ and has also been reported in paraspinal muscle degeneration in patients with isthmic spondylolisthesis^180^ and other degenerative spinal pathologies.^181^

Resident progenitor stem cells may also have roles in chondroid metaplasia in paraspinal tissues. These normally give rise to cells of a fibroblastic phenotype however they can also display osteogenic and chondrogenic potential. Chondroid metaplasia has also been observed in tendinopathy models through mechanical destabilization induced by partial transection of the infraspinatus tendon.^182^, ^183^, ^184^, ^185^ Perlecan expression is very significantly upregulated in an ovine rotator cuff tendinopathy model^116^ and aggrecan and ADAMTS expression is also disturbed in this model with the induced mechanical destabilization.^121^ Ovine IVDs subjected to surgically controlled annular incisions are also mechanically destabilized and the chondroid metaplasia we observed may be a consequence.
